# Infant Feeding Practices, Nutrition, and Associated Health Factors during the First Six Months of Life among Syrian Refugees in Greater Beirut, Lebanon: A Mixed Methods Study

**DOI:** 10.3390/nu14214459

**Published:** 2022-10-23

**Authors:** Joana Abou-Rizk, Theresa Jeremias, Lara Nasreddine, Lamis Jomaa, Nahla Hwalla, Jan Frank, Veronika Scherbaum

**Affiliations:** 1 Institute of Nutritional Sciences, Faculty of Natural Sciences, University of Hohenheim, 70599 Stuttgart, Germany; 2 Department of Nutrition and Food Sciences, Faculty of Agricultural and Food Sciences, American University of Beirut, Beirut 11-0236, Lebanon; 3 Department of Human Sciences, College of Health and Sciences, North Carolina Central University, Durham, NC 27707, USA or; 4 Faculty of Agricultural and Food Sciences, American University of Beirut, Beirut 11-0236, Lebanon

**Keywords:** early initiation of breastfeeding, exclusive breastfeeding, bottle feeding, anemia, nutrition and health aspects, infants under six months, Syrian refugees, Lebanon

## Abstract

The objective was to describe infant feeding practices, nutrition and related health aspects of infants under six months among Syrian refugees in Greater Beirut, Lebanon. A cross-sectional study was conducted among Syrian refugee mothers with infants under six months in July–October 2018 (N = 114). Additionally, eleven focus group discussions were conducted to explore supportive factors and barriers associated with early breastfeeding practices. The prevalence of pre-lacteal feeding was high (62.5%), whereas early initiation of breastfeeding was low (31%), and exclusive breastfeeding very low (24.6%). One-fifth of the infants were anemic (20.5%) and 9.6% were wasted. A significantly higher proportion of non-exclusively breastfed infants had a fever and took medicines than those who were exclusively breastfed. Supporting factors of adequate infant feeding practices comprised knowledge on maternal nutrition and exclusive breastfeeding, along with receiving support from healthcare professionals and family members. Identified barriers included preterm delivery, pre-lacteal feeding, an at-risk waist circumference and moderate to severe depression among mothers, bottle feeding, early introduction of food, maternal health reasons, breastmilk substitutes’ distribution, and misinformation offered by mothers-in-law. To address sub-optimal feeding practices documented among Syrian refugees, awareness on proper breastfeeding practices, maternal nutrition, and psychosocial support should be provided to mothers and family members alike.

## 1. Introduction

The benefits of breastfeeding have been well-documented in the long- and short-term for both the mother and child [1]. Human breast milk is renowned to be the safest and healthiest food for infants, offering invaluable protection against infections and their consequences [2,3]. As such, the World Health Organization (WHO) and United Nations International Children’s Emergency Fund (UNICEF) strongly recommend early initiation of breastfeeding within the first hour of birth and exclusive breastfeeding (EBF) for the first six months of life for optimal growth and development [4,5]. This is most critical during emergencies, as infants and children quickly fall among the most vulnerable victims. Malnutrition and infections emerged as the leading cause of childhood mortality and were often associated with crowded and unsanitary spaces subsequent to large-scale migration [6,7].

The global nutrition target was set to increase the rate of EBF in the first six months up to at least 50% by 2025 and 70% by 2030 [8,9]. Despite the improvement in the global prevalence of EBF from 36.5% in 2000–2009 to 45.7% in 2010–2018 in Low- and Middle-Income Countries, the Eastern Mediterranean region was among those that were the most away from global targets. Yet, it was the only region to experience a downward trend in the EBF prevalence by 5.3% and recorded the lowest EBF prevalence (34.5%) in 2010–2018 [10]. By the end of 2010, the Middle East and North Africa region had witnessed a wave of revolutions followed by many conflicts and wars in countries such as Tunisia, Egypt, Libya, Yemen, and Syria, with poverty and food insecurity being the main drivers and outcomes simultaneously [11].

The Syrian refugee crisis has become the largest worldwide with more than 6.7 million being forced to flee by the end of 2018. Lebanon hosted more than 1.5 million Syrian refugees, making it the country with the highest per capita concentration of refugees worldwide [12,13]. Syrian refugees in Lebanon have been subjected to harsh living conditions affecting their food security, nutritional status, and mental health, particularly among mothers and children [14,15,16]. Exposure to wars and conflicts could negatively impact maternal mental health and nutrition, resulting in long-lasting intergenerational effects on the health of the child. Evidence have shown that war times could decline breastfeeding rates and contribute to increased acute and chronic malnutrition among children [17,18].

Very few studies and reports have examined breastfeeding practices among Syrian refugees in Lebanon [19,20]. Up to date, no existing studies have examined early breastfeeding practices, the prevalence of anemia, and the nutritional status of infants under six months and their associated health factors among Syrian refugees in Lebanon. The objectives of this study were to (1) characterize infant feeding practices as well as anemia, nutritional status and related health aspects of infants under six months among Syrian refugees in Greater Beirut, Lebanon, (2) identify factors related to early initiation of breastfeeding, exclusive breastfeeding, and nutritional status of infants under six months, and (3) investigate barriers to the initiation of breastfeeding and exclusive breastfeeding among Syrian refugees with children under five years.

## 2. Materials and Methods

### 2.1. Study Design and Sampling Strategy

The mixed-methods survey was part of a larger project conducted among Syrian refugees living in the Greater Beirut area in Lebanon (July–October 2018). This area represented the urban agglomeration of the capital city of Beirut and its adjacent districts of Mount Lebanon Governorate, constituting the melting pot of the country. Primary health care centers were identified in localities with the highest level of vulnerability according to the United Nations Office for the Coordination of Humanitarian Affairs (UNOCHA) [21]. Mothers with at least one child aged less than five years were enrolled through primary health care centers using a two-step purposeful sampling strategy. The original sample size was calculated based on previous prevalence of anemia among Syrian refugee women in Lebanon to provide a power of 80%, a margin of error of 5% at 95% CI, and a design effect of 1.5 (N = 444). Mother-child pairs were eligible to participate if the inclusion criteria were met: (1) mother and child were Syrian, (2) the mother was between 15 and 49 years old, and (3) the child was aged 0–59 months and did not suffer from any inborn errors of metabolism or physical malformations. Out of the 590 eligible mother–child pairs to participate in this research project, 489 were recruited (17.1% non-response rate) and 433 completed the interview (11.4% dropout rate). Further details on the research project were presented elsewhere [15]. For this current study, mothers with children less than six months were included (N = 114).

### 2.2. Recruiting Strategy and Data Collection

In the original research project, mothers with children under five years were identified in primary health care centers via three approaches, including (1) the nurses at the centers, (2) direct contact by trained research assistants in the waiting rooms of the centers, and (3) posting flyers with a brief description of the project in the centers. An oral script was used by the research assistant to introduce the project to the potential participant, check the eligibility criteria, and seek the informed written consent of the mother.

One-on-one interviews were conducted by well-trained enumerators (Collaborative Institutional Training Initiative certified) to collect data using a multi-component questionnaire (July–September 2018). Data quality control and random checks were conducted during data collection and entry to increase the accuracy of the data and reduce the risk of reporting bias. Data were collected on socio-economic, household, maternal characteristics, nutritional status of mothers and their children, infant health and birth characteristics, infant feeding practices, maternal dietary diversity, and maternal mental health. The minimum dietary diversity for women (MDD-W) of reproductive age was measured using the open recall method. Achieving MDD-W was defined as consuming five or more out of ten food groups [22]. The Patient Health Questionnaire-9 (PHQ-9) was used to measure depression among mothers [23]. Self-reported gestational age, weight, and length at birth were recorded. Birth weight was classified as low birth weight (<2500 g) [24], normal birth weight (2500–3999 g), and macrosomia (≥4000 g) [25]. Gestational age of infants was collected in months instead of weeks for cultural adaption and was classified as: pre-term for infants born before 37 weeks (<nine months) and full-term from 37 weeks (≥nine months) [26]. The household monthly income was classified according to the legal minimum wage in Lebanon, approximately equal to 750,000 Lebanese Pounds (LBP) (equivalent of USD 500 at the time of data collection) [27]. Crowding index was based on the American Crowding Index definition (total number of co-residents by number of rooms without kitchens, bathrooms, hallways, balconies, and garages) [28].

### 2.3. Definitions of Infant Feeding Practices

Data on infant feeding practices were collected using a culturally adapted questionnaire based on the 2008 Infant and Young Child Feeding (IYCF) indicators [29,30]. Definitions of infant feeding indicators used in this study were described as follows:

1. Ever breastfed (EvBF): percentage of infants who ever breastfed successfully after birth (Indicator 1) [31].
2. Ever received pre-lacteal feeding before any breast milk: percentage of infants who were offered pre-lacteal food or liquid before receiving any breast milk after birth.
3. Early initiation of breastfeeding: percentage of infants who were put to the breast within one hour of birth (Indicator 2) [31].
4. Child breastfed yesterday: percentage of infants who received breast milk yesterday, including drops or syrups and anything else (any food or liquid, non-human milk, and formula) [29].
5. Bottle feeding yesterday (BoF): percentage of children who were fed from a bottle with a nipple during the previous day (Indicator 16) [31].
6. Ever received infant formula and/or other types of milk: percentage of infants who ever received infant formula and/or other types of milk.
7. Introduction of solid, semi-solid, or soft foods before six months: percentage of infants who ever received solid, semi-solid, or soft foods before six months.

In addition, a detailed breakdown and description of infant feeding practices among infants under six months during the previous day (adding up to 100%) was defined as follows:

8. Exclusive breastfeeding: percentage of infants who received exclusively breastmilk yesterday, including drops or syrups, but without anything else (Indicator 3) [31].
9. Breastfeeding, mixed with infant formula, without other liquids and/or solid foods: percentage of infants who received breast milk and infant formula (excluding animal milk, other liquids and foods) (adapted from Indicator 5) [31].
10. Breastfeeding, with other liquids and/or solid food: percentage of infants who received liquids (animal milk, tea, yogurt, sugary water, etc.) and solid foods in addition to breast milk, but without infant formula.
11. Mixed milk feeding, with other liquids and/or solid foods: percentage of infants who received infant formula, any food, and other liquids yesterday, in addition to breast milk (adapted from Gao et al., 2016) [32].
12. Exclusive infant formula feedings, with other liquids and/or solid foods: percentage of infants who received infant formula yesterday, animal milk, other liquids, and solid foods, but without any breast milk (adapted from Gao et al., 2016) [32].

### 2.4. Anthropometric and Biochemical Assessment

Standardized techniques and calibrated equipment were used to measure anthropometrics among mothers and infants. The average of two measurements was recorded to the nearest decimal. The waist circumference of non-pregnant mothers was measured with light clothing using a non-elastic measuring tape (SECA 201) and was classified as normal (≤79 cm) and at-risk (>80 cm) [33]. As for infants under six months, weight and length were measured using a measuring mat (SECA 417) and an electronic 2-in-1 weighing scale (SECA 876). Their nutritional status was defined using the WHO child growth standards and the WHO Anthro Survey Analyzer to derive the *z*-scores [34]. Stunting was defined using the length/height-for-age Z-scores (L/HAZ < −2), underweight using the weight-for-age Z-scores (WAZ < −2), wasting using the weight-for-length/height Z-scores (WHZ < −2), and overweight/obese using the body mass index-for-age (BMI-for-age) Z-scores (BAZ > +2) [35]. Microcephaly was assessed using the head circumference Z-scores (HCZ < −2) [36]. Mid-upper arm circumference (MUAC) was measured using the UNICEF MUAC measuring tapes and was classified as acute malnutrition (<115 mm) and at-risk malnutrition (115–129 mm) [37]. Anemia status was assessed among infants under six months by certified phlebotomists using the “HemoCue Hb301 System” to measure hemoglobin (Hb) concentrations. Given the lack of WHO criteria for classifying the severity of anemia for this age group [38], total anemia was defined as Hb < 10.5 g/dL, according to Marques et al. (2014) [39].

### 2.5. Statistical Analysis

Descriptive analyses were presented as frequencies with percentages for categorical variables and as means with standard deviations (SD) for continuous variables. Characteristics between non-exclusively and exclusively breastfed infants were conducted using chi-square analysis for categorical variables and independent sample t-test to compare means across groups. Factors associated with early initiation of breastfeeding, time of breastfeeding initiation, and exclusive breastfeeding (as dichotomous dependent variables) were identified using multiple logistic regressions. Factors associated with the weight-for-length/height Z-score (as continuous dependent variables) was evaluated using a multiple linear regression. Different models were used for each of the dependent variables. All variables that have shown a *p*-value < 0.05 in the univariate logistic or linear regressions were checked for multicollinearity using the tolerance, variance inflation factors (VIF), and the condition index prior being included in the models. Independent variables that were entered in the models were reported. The significance of regression models was evaluated using R-squared, the overall percentage, and Hosmer and Lemeshow test for logistic regression models and using R-square and the normal probability plot of residuals for linear regression models. Results from the logistic regressions were expressed as aOR for adjusted odds ratios with a 95% confidence interval (CI) and from the linear regressions as aβ for adjusted β with a 95% CI. A *p*-value < 0.05 was considered statistically significant. KoBo ToolBox (version March 1, 2018, Harvard Humanitarian Initiative, Cambridge, MA, USA) was used for data entry and Statistical Package for Social Sciences (version 27.0, SPSS Inc., Chicago, IL, USA) was used to conduct the statistical analysis [40].

### 2.6. Qualitative Study and Analysis

A qualitative research approach was adopted to explore perceived barriers to, and facilitators of, infant feeding practices in relationship with maternal mental health and nutritional status, infant formula use, and traditional practices among Syrian refugee mothers living in Greater Beirut, Lebanon. A topic guide was developed and approved by the ethical board prior the onset of the study.

A pool of participants was drawn from the parent study by contacting mothers who had provided permission to be contacted for future research (n = 201). Overall, 183 mothers were invited to participate in focus group discussions (FGDs), of which 30 mothers partook together with 13 Syrian female relatives (sister, mother, or mother-in-law). In total, 43 Syrian women took part in 11 FGDs between September and October 2018. An additional informed consent was obtained from participants prior to the start of FGDs. Participants were randomly assigned to the FGDs, depending on their availabilities. Discussions were conducted in colloquial Arabic and lasted between 20 and 75 min, with two to nine participants per group, and were recorded using a digital voice recorder. Data collection was completed when data saturation was achieved for the studied themes.

FGDs were first transcribed to Latin Arabic and then translated to English. Transcripts were analyzed using MAXQDA software (version 2022.2, VERBI, Berlin, Germany) [41]. Qualitative content analysis was conducted using a combined technique of deductive and inductive thematic analysis. A coding guideline was developed with anchor quotes defining main and sub-themes. To meet the criteria of validity and reliability, a countercheck was conducted by trained members of the research team. Inter-code reliability checks were carried out to reduce inter-subjectivity by checking the coding guidelines and their corresponding quotes [42,43].

## 3. Results

Household and maternal characteristics are described in Table 1. Nearly half of the mothers were less than 25 years old (44.7%), had one to two children aged less than five years (53.1%), and lived with their extended family (51.8%) with a mean crowding index of 3.8 (±1.6). The majority were registered as refugees with the United Nations High Commissioner for Refugees (UNHCR) (82.3%) and had a household monthly income below the minimum wage (63.3%). Two-thirds of the mothers did not achieve MDD-W (64.0%), and three-quarters of non-pregnant mothers had an at-risk waist circumference (75.5%).

Table 2 presents feeding practices of infants under six months. The vast majority were ever breastfed (98.2%). Among those, only 31.0% were breastfed within the first hour of birth and 30.1% during the first day (1–23 h), while more than a third received late breastfeeding initiation (≥24 h; 38.9%). In addition, nearly two-thirds received pre-lacteal feedings before the onset of lactation (62.5%). The most common type of pre-lacteal feeding was infant formula milk (70.0%), followed by sugary water (31.4%) and herbal infusions (15.7%). Current feeding practices showed that most of the infants breastfed the previous day (90.4%) and almost half were bottle fed the day before (44.7%). Only 24.6% were exclusively breastfed during the previous day. Most of the infants received breast milk with other liquids or foods, but without infant formula milk (37.7%), followed by those who received breast milk and infant formula milk, with other liquids or foods (15.8%) or without other liquids or foods (12.3%). The most common types of liquids consumed the previous day included plain water (51.8%), infant formula milk (36.8%), and herbal infusions (10.5%). Furthermore, only 39.3% of the infants never received infant formula or other types of milk, and 25.9% received them since the first day of birth. In addition, 83.9% had never received solid, semi-solid, or soft foods before six months.

Table 3 displays maternal, birth, and health characteristics of infants according to their exclusive breastfeeding status. Overall, 79.1% of infants were born full-term (≥37 weeks), the prevalence of low birth weight (<2500 g) was 10.9%, and the rate of caesarean section reached 33.3%. The prevalence of anemia among infants was 20.5% and of wasting 9.6%. Mild and moderate to severe depression rates among mothers reached 17.6% and 15.7%, respectively. Among non-exclusively breastfed infants as compared to exclusively breastfed infants, a significantly higher proportion of Caesarean section (38.4% vs. 17.9%, *p* = 0.045), late breastfeeding initiation (≥24 h; 44.7% vs. 21.4%, *p* = 0.019), and maternal depression rates (mild: 21.7% vs. 4.0%, *p* = 0.032; moderate to severe: 18.1% vs. 8.0%, *p* = 0.032) were found.

In addition, a higher proportion of mothers attended one or less antenatal care visits (26.2% vs. 18.5%, *p* = 0.014) among non-exclusively breastfed infants compared to those who were exclusively breastfed. While nearly one third of the infants suffered from various symptoms in the past two weeks, a higher proportion of non-exclusively breastfed infants had a fever compared to exclusively breastfed infants (25.0% vs. 7.1%, *p* = 0.043). Likewise, significantly more non-exclusively breastfed infants were taking medicines in the past two weeks (46.8% vs. 23.1%, *p* = 0.033), particularly pain killers and anti-inflammatory tablets (67.6% vs. 16.7%, *p* = 0.018), than exclusively breastfed infants.

Factors associated with early initiation of breastfeeding, time of breastfeeding initiation, exclusive breastfeeding, and wasting using regression models are shown in Table 4. Mothers who initiated breastfeeding within the first hour of birth had significantly higher odds of not achieving MDD-W (aOR = 5.52, 95% CI:1.28–20.75) and having a higher WHZ score among their infants (aOR = 1.58, 95% CI: 1.03–2.43), compared to mothers who did not. On the other hand, early initiation of breastfeeding was significantly associated with lower odds of having a preterm birth (aOR = 0.18, 95% CI: 0.03–0.98), receiving pre-lacteal feeding among infants (aOR = 0.06, 95% CI: 0.02–0.20), and having an at-risk waist circumference (aOR = 0.19, 95% CI: 0.05–0.82), compared to those with late initiation of breastfeeding (>1 h after birth). Further analysis showed that late initiation of breastfeeding (≥24 h) was nearly 4-times more likely to occur among mothers with moderate to severe depression (aOR = 3.46, 95% CI: 1.09–10.97) as compared to those who initiated breastfeeding within the first day of birth. Exclusive breastfeeding was negatively associated with the age of the infant (aOR = 0.56, 95% CI: 0.39–0.81) and bottle feeding (aOR = 0.04, 95% CI:0.00–0.38), compared to those who were not exclusively breastfed. As for wasting, the WHZ score was positively associated with receiving infant formula milk (aβ = 1.41, 95% CI: 0.34–2.48) and negatively with bottle feeding (aβ = −0.83, 95% CI: −1.52; −0.14).

Findings from FGDs related to infant feeding practices are found in Table 5 with major themes, codes, and representative quotes. Two major thematic axes emerged as follows: (1) Enablers and (2) Barriers for early initiation of breastfeeding and exclusive breastfeeding.

Under the Enablers axis, two major themes were generated representing (1.a) knowledge on infant feeding practices and maternal nutrition and (1.b) support from healthcare providers and family. Breastfeeding emerged as a core practice according to their cultural beliefs as mothers referred to it as a “*right*” (Mother 3) and crucial for the development of the child. Mothers highlighted the importance of proper maternal nutrition and to be able to “*eat everything […] and not stop anything*” (Mother 5). Many mothers also demonstrated positive attitude towards exclusive breastfeeding and eagerly confirmed to have given their infants “*only breast milk, until six months (…), not even water*” (Mother 4). Receiving support from healthcare professional or family was discussed by participants. A couple of mothers mentioned that they follow the recommendations of doctors, yet they still valued their family members’ advice. As a result, several women preferred to talk to their mothers or mothers-in-law first and turn to a doctor when “*the condition progresses*”. When feeling overwhelmed, one mother stated that she took the advice of her sister-in-law to “*calm down*” because she “*can’t breastfeed*” and other times she would call her mother “*to take information from her*” (Mother 10). Other mothers stated that “*we all breastfeed*” (Mothers 12 and 13), indicating that it was a common practice adopted well and supported by their families and communities.

As for the Barriers axis, four major themes emerged, including (2.a) pre-lacteal feeding, (2.b) infant feeding practices, (2.c) maternal health reasons, and (2.d) social factors. According to the discussions with the mothers, pre-lacteal feedings and early introduction of solid, semi-solid, or soft foods may have not been seen as the main barrier to early initiation of breastfeeding and exclusive breastfeeding. Pre-lacteal feeding consisted of oral rehydration solutions and possibly infant formula milk, as mothers referred to it as “*a small box [referring to a type of feed or solutions]*”, and mothers seemed to be content with using it until “*the milk came in*” (Mother 14). Similarly, early introduction of foods appeared to be common and was perceived as a practice that encouraged infants to taste a variety of food “*so they won’t be disgusted by it when they grow up*” (Mother 1). Mothers appeared to be satisfied with introducing a “s*mall quantity*” of foods such as family dishes, “*yogurt, fig jam, herbal tea*”, “*fruits*”, and “*mashed rice*” at “*five months*” or even earlier starting “*three to four months*” (Mothers 1, 4, 15, 16, and 17).

When asked about the use of infant formula milk, many mothers confirmed having used a mixed milk feeding to “*help*” the child for various reasons “*from the first month*” or between the second and fourth months (Mothers 4, 17, and 18). Water consumption was also widely discussed. It appears that this practice was known among mothers “*from day 40 [after birth]*” (Mother 18). As for herbal tea consumption, one mother explained to have used it as an alternative to breast milk because she “*didn’t have any milk left to breastfeed*”, which caused her child to be hospitalized because of “*so much herbal tea*” (Mother 3).

On the other hand, discussions suggested that maternal health reasons were perceived as a major barrier to adequate breastfeeding practices. Perceived lack of breast milk was widely mentioned as a reason for not breastfeeding. Commonly used statements included “*I didn’t have any breast milk*”, “*it was dry*”, and “*the milk stopped*” (Mothers 1, 3, 19, and 20). Some mothers even highlighted that they “*had no milk; it was dry from lack of nutrition*” (Mother 10), indicating insufficient maternal dietary intake was a known barrier to breastfeeding. Furthermore, mothers were able to express that having poor mental health, an illness, or feeling tired were known obstacles to proper breastfeeding practices. One mother readily stated that “*[when I am worried or tired], I can’t breastfeed (…)*” (Mother 10) and “*I’m getting tired when I’m breastfeeding, I’m very tired, my bones, they hurt. (…) I can’t stand up, when I finish breastfeeding, I feel beaten and dizzy…*” (Mother 3). Complications during pregnancy or delivery and premature delivery were also viewed by mothers as barriers to breastfeeding. Mothers openly shared their struggles to initiate breastfeeding because of experiencing “*complications*” and “*bleeding*” during delivery (Mother 23) or having a “*very small*” newborn (Mother 25).

As for social factors, discussions indicated that the use of breastmilk substitutes seemed to influence the mother’s choice to initiate or continue breastfeeding. Several mothers readily acknowledged receiving free samples of infant formula milk from healthcare professionals or local non-governmental organizations. Mothers also stated receiving strong recommendations to use infant formula milk from healthcare professionals when their infants were very ill or when “*there wasn’t any milk*” (Mother 13). In addition, discussions revealed that mothers were aware of some misinformation being passed down by earlier generations, particularly from mothers-in-law. For instance, mothers shared that they received specific nutritional advice to avoid or consume a certain food group such as “*potato and rice only*” (Mothers 20 and 6) and for infants to consume “*starch*” (Mother 20), resulting in malnutrition among both the mother and her infant.

## 4. Discussion

This is the first study to investigate infant feeding practices as well as the prevalence of anemia and the nutritional status of infants under six months among Syrian refugees in Greater Beirut, Lebanon—representing an urban setting of a humanitarian crisis. Despite the very high rate of ever breastfed infants (98.2%), less than a third were breastfed within the first hour of birth (31.0%) and a quarter were exclusively breastfed (24.6%) in our study. Our prevalence was comparable to those reported in Syria in 2019 [44,45] and among Syrian refugees in Lebanon in 2013 [19], but lower than those recorded in Syria before the start of the war in 2009–2010 [44,45] and among Syrian refugees in Southern Turkey, Northern Lebanon, and Jordan in 2016–2020 [20,46,47]. Similarly, low exclusive breastfeeding (EBF) rates were documented among internally displaced persons in eastern Ukraine [48] and Sahrawi refugees in Algeria [49]. Our findings were also in in line with recent national and regional studies in Lebanon reporting a prevalence of EBF varying from 30% to 59% among children under six months conducted in 2019–2021 [50,51,52]. However, our rates remained below the global and Middle East and North Africa regional rates of early initiation of breastfeeding and EBF in 2014–2020 [44,45].

Ever breastfeeding was most widespread among Syrian refugees in Greater Beirut, Lebanon. Similar findings were registered among Syrian refugees in Northern Lebanon and Turkey [20,46,53]. In our study, mothers explained that breastfeeding is a core practice in their culture as they “*all breastfed*” and believed that breastfeeding is a “*right*” for their children. Breastfeeding intention was found to have a crucial role in the initiation and sustainability of effective breastfeeding among refugees [54,55]. According to the World Health Organization (WHO), ever breastfeeding is a reflection of the acceptance of breastfeeding in the culture [31]. In fact, the prevalence of ever breastfeeding, early initiation, and EBF were even higher in Syria in 2009–2010 before the start of the war [44,45], indicating that this practice has been well integrated in their culture. Nevertheless, evidence have shown that breastfeeding practices deteriorate during humanitarian crises and conflicts, increasing the risk of infant malnutrition [48,56].

The prevalence of anemia (20.5%) and wasting (9.6%) among children under six months were classified as a medium public health significance in our study [57,58]. Very few studies examined anemia levels of infants under six months in the literature, especially among refugees. The anemia level in our study were not too far from findings recorded in Argentina (28.9%) [59] and South Africa (33%) [60]. As for wasting, recent surveys conducted among Syrian refugees and Lebanese children aged 6 to 59 months in Lebanon showed that childhood wasting was maintained to about 5% in 2018–2021 [15,52]. However, a similar prevalence of wasting was found among infants of Sahrawi refugees [49]. This suggests that infants among the refugee population may be at an increased risk of acute malnutrition from an early age.

Pre-lacteal feedings were given to 62.5% of the infants in our study, with the majority receiving infant formula milk, followed by sugary water and herbal infusions, before lactation was started. It appears that mothers in our study did not view pre-lacteal feedings, consumption of water or herbal tea, and early introduction of foods as a barrier to exclusive breastfeeding. Syrian refugees in Turkey and Jordan as well as Sahrawi refugees in Algeria practiced similar customs [47,49,61]. For instance, sugary water was believed to cleanse the intestines and prevent jaundice by most parents and family members in Syria [61]. Other common beliefs were observed among internally displaced persons in eastern Ukraine, such as giving infants water when the mother felt thirsty while it was warm outside [48]. Some mothers also introduced foods before the age of six months, despite the WHO recommendations. In our study, mothers justified this common practice as an approach to get their children to eat “*everything*” and “*anything*” at home. Others believed that introducing food early would lead to a chubby baby, as a sign of good health [62]. Similar traditions were also documented among Syrian refugees in Turkey [61], Lebanon [20], and Germany [62] as well as refugees in Algeria [49] and displaced persons in eastern Ukraine [48].

Early initiation of breastfeeding was shown to be protective against receiving pre-lacteal feeding and wasting (WHZ) among infants. Timely onset of breastfeeding was also significantly associated with EBF, despite the lack of association in the regression models. Breastfeeding within the first hour of birth is known for its decisive role in securing that newborns receive colostrum feedings, limiting the possibilities of feeding newborns anything other than breast milk, and establishing EBF successfully [5]. This is confirmed by the robust literature supporting the advantages of breastfeeding for the baby by reducing gastrointestinal and respiratory infections and non-communicable diseases as well as increasing intelligence in the long-term [63,64,65]. Our findings also showed that a significantly higher proportion of non-exclusively breastfed infants suffered from a fever and received medicines compared to those who were exclusively breastfed. Moreover, early introduction of complementary food has been known to shorten the duration of breastfeeding and stop it prematurely [66]. Therefore, adequate infant feeding practices can set the child on the right path to prevent malnutrition from early infancy [67].

Bottle feeding was also identified as a main barrier to exclusive breastfeeding in our study. In addition, the use of bottles had a significant negative association with the weight-for-length/height Z-score, while the use of infant formula milk had a positive association. According to the WHO, feeding bottles and teats were discouraged as they are often difficult to keep clean and could lead to the transmission of pathogens. Their uses increased the risk of diarrhea, dehydration, and malnutrition [6]. Bottle feeding was also known to increase unfavorable behaviors during breastfeeding, such as suckling behavior, affectivity, baby’s response, and the mother/baby position, which might interfere with the infant’s weight gain and increase the risk of early weaning [68]. Hence, it is recommended to use a feeding cup when needed and to secure a suitable breastmilk substitute, prepared according to instructions for safe preparation and use, to be given only to infants who do not have access to breast milk [7]. The prevalence of bottle feeding documented in our study (44.7%) was similar to those observed among the Lebanese population and Syrian refugees in Lebanon [19,51], but lower compared to Syrian refugees in Jordan [47]. Our discussions with the mothers indicated that the use of breastmilk substitutes seemed to influence the mother’s choice to breastfeed due to free samples of infant formula milk distributed or strong recommendations for their use by healthcare professionals. These findings were a direct reflection of the violations to the Code of Marketing of Breastmilk Substitutes previously documented in Lebanon [69,70].

Moreover, having a pre-term delivery was found to be negatively associated with early initiation of breastfeeding. Studies have shown that preterm delivery might delay the initiation of lactation among mothers [71]. Nevertheless, it would still be possible to establish exclusive breastfeeding among preterm infants [72]. A high prevalence of caesarian delivery was recorded in our study (33.3%), exceeding the global rate of 21.1% [73]. Caesarian delivery, labor complications, and premature birth also emerged as barriers to breastfeeding during focus group discussions in our study. Caesarian deliveries are known to hamper the initiation of breastfeeding and exclusive breastfeeding rates. This might be mediated by various reasons, including the delayed onset of breastfeeding and disrupted mother-infant interaction due to postoperative care routines [74]. The lack of association of caesarian delivery and breastfeeding in our study might be explained by the large number of caesarian sections performed that could have been elective and not medically recommended. As a result, mothers who underwent elective caesarian sections might have better outcomes and be able to start breastfeeding earlier compared to those who had a medical reason [75]. Mixed findings on the role of caesarian sections as a predictor of delayed initiation of breastfeeding were also documented in Lebanon [75,76].

Low dietary diversity among mothers was also associated with higher odds of early initiation of breastfeeding. Poor dietary diversity was also linked to low income and food insecurity among Syrian refugee mothers in Greater Beirut, Lebanon [16]. This suggests that mothers facing increased economic vulnerabilities would be more inclined to initiate breastfeeding early as breast milk is known to be more cost-effective. In addition, Syrian refugees in Turkey and Lebanon stated that breast milk is “*free*”, “*less costly and more natural*” [53,61]. However, it was also mentioned by the mothers that insufficient maternal nutrition and poor health were major reasons to halt breastfeeding prematurely. Among Syrian refugees in Greater Beirut, Lebanon, lactating mothers were found to have higher proportions of nutritional inadequacies and 19.4% of them suffered from anemia [15]. In addition, Syrian refugees in Turkey reported that malnutrition and fatigue of mothers were often the biggest obstacles to breastfeeding [61]. These findings shed the light on the role of adequate maternal nutrition during pregnancy and lactation as it impacts the development and health of the offspring [77,78], which was particularly difficult to achieve among Syrian refugee mothers in Greater Beirut, Lebanon [15,16].

Maternal-related adverse factors also included maternal obesity and maternal depression. Having an at-risk waist circumference was associated with lower odds of early initiation of breastfeeding. This is concerning as more than 60% of the mothers in our study were found to be overweight or obese [15]. According to the literature, maternal obesity is associated with lower rates of early initiation of breastfeeding and EBF not only due to mechanical factors, hormonal imbalances, and a delayed onset of lactogenesis II, but also to psychosocial factors associated with body image dissatisfaction or concerns [79,80]. Maternal depression was also identified as a predictor to delayed initiation of breastfeeding. Possible explanations are that maternal psychological distress, such as depression or anxiety, may impair the release of oxytocin and delay the onset of lactogenesis, thus reducing breastfeeding outcomes [81]. Crowded spaces were also reported to negatively impact breastfeeding practices among Syrian refugees in Lebanon [53]. This could be explained by the strong association between poor maternal mental health, food insecurity, and a high crowding index found among Syrian refugee mothers in Lebanon [16]. Particularly, stress related to conflicts was recognized as a major contributor to the discontinuation of breastfeeding among refugees and displaced persons, as it might interfere with the mother’s let down reflex and lactation [6,46,48,53]. These cues could be easily perceived as a sign of insufficient milk production by mothers [6], as stated during focus group discussions. Providing reassurance and support to these mothers is beneficial and would enhance the lactation outcome [6], particularly among mothers that were experiencing loss of social support due to migration [61]. Mothers in our study described that they were able to breastfeed following the advice of a family member to “*calm down*” when worried or tired. These findings emphasize the significance of the role of psychosocial support for effective breastfeeding practices.

Receiving support from healthcare providers and family members were mentioned as enablers for breastfeeding by mothers in our study. Spousal support was found to be a strong influencer for breastfeeding attitudes [54], along with female relatives which were viewed as key sources of support for breastfeeding among Syrian refugee mothers in Turkey, Germany, and Lebanon [53,61,62]. However, some nurses and midwives reported that the support provided by grandmothers was sometimes seen as excessive since it reduced the mother’s contact with the newborn and affected the mother–infant bonding [61]. In addition, some mothers also discussed receiving misinformation from their mothers-in-law that lead to malnutrition and an early cessation of breastfeeding in our study. This indicates the importance of engaging family members such as grandmother and mothers-in-law in counselling, programs, or interventions targeted to promote optimal infant and young child feeding practices as well as maternal nutrition [82]. Although nutrition knowledge is known to be a key modulator in breastfeeding practices [82,83], a strong intention to breastfeed prenatally and a positive attitude towards breastfeeding are also significant predictors [84]. Furthermore, the number of antenatal care visits was positively associated with exclusive breastfeeding, even though this relationship was not significant in the multivariate models. The positive impact of antenatal care visits on breastfeeding practices has been well documented in the literature [6,85,86]. However, the lack of or difficulty to access antenatal care might act as a barrier for Syrian refugee mothers, especially among those not registered as refugees with the UNHCR in Lebanon or those facing a language barrier such as in Turkey and Germany [15,46,61,62].

Findings of this study should be interpreted considering some limitations and strengths. This is a cross-sectional study, hence limiting the ability to infer causality. Data collection was focused on the most vulnerable areas of Greater Beirut using a purposeful sampling approach; thus, findings cannot be generalized to rural areas or the whole country. However, one strength of our study is the inclusion of unregistered refugees living in the catchment area of these primary health care centers, which would have not been possible if a randomized approach using the UNHCR list of refugees was used. Another limitation is the use of the previous day’s feeding to measure the proportion of exclusively breastfed infants. The proportion of EBF can be overestimated as some infants may have not received other liquids or foods on the day before the interview [87]. It is also worth noting that data on birth characteristics may be subjected to recall bias as the gestational age, weight, and length at birth were self-reported. In addition, the definition of gestational age was adapted to the culture and was interpreted in months instead of weeks as understood by the mothers. Therefore, the proportion of full-term births could be overestimated. Lastly, the Patient Health Questionnaire-9 (PHQ-9) was validated among Lebanese adults instead of Syrian refugees. It also had a poor specificity in capturing depressive symptoms, yet a good sensitivity. Thus, it was regarded as a useful screening tool for depression in settings lacking sufficient psychiatric care [88].

## 5. Conclusions

In conclusion, this study demonstrated poor infant feeding practices among infants under six months of Syrian refugees in Greater Beirut, Lebanon. Our findings showed that 20.5% and 9.6% of infants under six months were anemic and wasted, respectively. Despite a nearly universal onset of breastfeeding, early initiation and exclusive breastfeeding were considerably low. While the early initiation of breastfeeding was associated with lower odds of receiving pre-lacteal feeding and wasting among infants, bottle feeding was identified as a significant obstacle to exclusive breastfeeding. Key maternal-related factors were found to be adversely related to the early initiation of breastfeeding, which included low dietary diversity, obesity, and depression. The role of family members’ support, antenatal and post-natal care, and nutrition education for effective breastfeeding practices were underlined in our study.

Our findings could help strengthen existing campaigns and interventions to address the identified gaps and barriers. Raising awareness on proper breastfeeding practices would be essential for mothers and their family members alike. Psychosocial support would be particularly important for refugee mothers, considering the role of maternal mental health status in the successful establishment of breastfeeding. As maternal nutrition and infant nutrition are tightly linked, nutrition specific interventions should focus their efforts on improving the nutritional status of mothers at the community and individual levels to reduce infant malnutrition. Furthermore, the implementation of the Code of Marketing of Breastmilk Substitutes and the Baby Friendly Hospital Initiative would need to be reinforced in Lebanon. Future research should investigate the determinants of anemia and the nutritional status among infants under six months in prospective cohort studies.

## Figures and Tables

**Table 1:** Socio-economic, maternal characteristics, and child growth indicators of infants under six months among Syrian refugees in Greater Beirut, Lebanon.

Variables ^a^	Total (N = 114)	Variables ^a^	Total (N = 114)
Socio-Economic and Household Characteristics		Maternal Characteristics	
Sex of the child Male Female	62 (54.4) 52 (45.6)	Age of the mother (years) <25 years 25 to 29 years ≥30 years	26.40 ± 5.7 51 (44.7) 33 (28.9) 30 (26.3)
Mother’s education level No schooling/Illiterate Primary, Intermediate school Secondary school and higher	19 (16.7) 68 (59.6) 27 (23.7)	MDD-W for mothers Not achieved (<5 food groups) Achieved (≥5 food groups)	73 (64.0) 41 (36.0)
Father’s education level No schooling/Illiterate Primary, Intermediate school Secondary school and higher	17 (15.2) 76 (67.9) 19 (17.0)	Waist circumference of non-pregnant mothers (n = 110) ^b^ Normal (≤79 cm) At-risk (>80 cm)	27 (24.5) 83 (75.5)
Mother’s employment status No paid job/Housewife Paid job (daily/part-/full-time)	109 (98.2) 2 (1.8)	**Child growth indicators (Z-scores)**	
Father’s employment status No job Part-time job Full-time job/ Self-employed	5 (4.5) 50 (45.5) 55 (50.0)	Length/Height-for-age	0.60 ± 2.3
Monthly household income ≤750,000 LBP >750,000 LBP	69 (63.3) 40 (36.7)	Weight-for-age	0.09 ± 1.8
Crowding index score	3.77 ± 1.6	Weight-for-length/height	−0.46 ± 1.4
Length of stay in Lebanon (years)	3.90 ± 4.5	Body Mass Index-for-age	−0.33 ± 1.4
UNHCR refugee registration status No Yes	20 (17.7) 93 (82.3)	Head-circumference-for-age	0.19 ± 1.9
Household type Nuclear family Extended family	55 (48.2) 59 (51.8)	Mid-upper arm circumference-for-age	0.34 ± 1.3
Number of children < 5 years per household 1 to 2 3 to 4 ≥5	2.53 ± 1.4 60 (53.1) 43 (38.1) 10 (8.8)		

LBP: Lebanese Pounds; UNHCR: United Nations High Commissioner for Refugees; MDD-W: Minimum Dietary Diversity for Women; ^a^ Categorical variables are expressed as n (%) and continuous variables are expressed as mean ± SD. Lack of corresponding sum of frequencies with total sample size is due to missing data. ^b^ Non-pregnant mothers include lactating and non-pregnant non-lactating mothers.

**Table 2:** Feeding practices of infants under six months among Syrian refugees in Greater Beirut, Lebanon (questionnaire-based).

Infant Feeding Practices during the Previous Day	Total (N = 114)	Ever Applied Infant Feeding Practices	Total (N = 114)
**Child breastfed the previous day** Yes No	103 (90.4) 11 (9.6)	**Ever breastfed**	112 (98.2)
**Bottle feeding yesterday** Yes No	51 (44.7) 63 (55.3)	**Ever received pre-lacteal feeding before any breast milk**	70 (62.5)
**Detailed description of feeding practices**		Types of pre-lacteal feeding received (n = 70) *	
Exclusive breastfeeding	28 (24.6)	Infant formula milk	49 (70.0)
Breastfeeding, mixed with infant formula, without other liquids and/or solid foods	14 (12.3)	Sugary water	22 (31.4)
Breastfeeding, with other liquids and/or solid food	43 (37.7)	Herbal infusions	11 (15.7)
Mixed milk feeding, with other liquids and/or solid foods	18 (15.8)	Bottled/boiled water	3 (4.3)
Exclusive infant formula feeding, with other liquids and/or solid foods	11 (9.6)	Other liquids/solid foods (e.g., yogurt and electrolytes)	2 (2.9)
Types of liquids consumed the previous day *		**Initiation of breastfeeding**	
Plain water	59 (51.8)	≤1 h	35 (31.0)
Infant formula milk	42 (36.8)	1–23 h	34 (30.1)
Herbal infusions (e.g., anis, chamomile, caraway)	12 (10.5)	≥24 h	44 (38.9)
Other milk (tinned, powdered, or fresh)	7 (6.1)	**Ever received infant formula and/or other types of milk**	70 (61.4)
Yogurt	7 (6.1)	**Age of introduction**	
Juice	6 (5.3)	Never	44 (39.3)
Clear broth	1 (0.9)	≤1 day	29 (25.9)
Other liquids (e.g., sodas, chocolate drinks)	1 (0.9)	2–29 days	14 (12.5)
		1–3 months	19 (17.0)
		4–5 months	6 (5.4)
		Milk types introduced (n = 68) *	
		Infant formula milk	62 (91.2)
		Powdered cow’s milk	5 (7.4)
		Fresh cow’s milk	1 (1.5)
		**Introduction of solid, semi-solid, or soft foods before six months**	18 (15.8)
		**Age of introduction**	
		Never	94 (83.9)
		<3 months	3 (2.7)
		4 months	5 (4.5)
		5 months	10 (8.9)

Categorical variables are expressed as n (%). Lack of corresponding sum of frequencies with total sample size is due to missing data. * Multiple answers were allowed.

**Table 3:** Maternal, birth, health characteristics, anemia, and nutritional status of infants under six months by exclusive breastfeeding status among Syrian refugees in Greater Beirut, Lebanon.

Variables ^a^	Exclusive Breastfeeding		Total	*p*-Value ^b^
	Yes	No		
Birth Characteristics	n = 28	n = 86	N = 114	
Type of delivery Vaginal birth Caesarean section	23 (82.1) 5 (17.9)	53 (61.6) 33 (38.4)	76 (66.7) 38 (33.3)	**0.045**
Self-reported gestational age (months) Preterm (<37 weeks) Full-term (≥37 weeks)	8.85 ± 0.6 4 (15.4) 22 (84.6)	8.95 ± 0.3 19 (22.6) 65 (77.4)	8.92 ± 0.4 23 (20.9) 87 (79.1)	0.468 0.428
Self-reported weight of child at birth (kg) Low birth weight (<2500 g) Normal birth weight (2500–3999 g) Macrosomia (≥4000 g)	3.13 ± 0.5 3 (10.7) 23 (82.1) 2 (7.1)	3.09 ± 0.6 9 (11.0) 70 (85.4) 3 (3.7)	3.10 ± 0.6 12 (10.9) 93 (84.5) 5 (4.5)	0.750 0.747
Self-reported length of child at birth (cm)	48.00 ± 6.9	50.37 ± 2.7	49.6 ± 4.5	0.201
**Maternal and child characteristics**				
Age of the infant on the day of the survey <4 months ≥4 months	2.24 ± 1.0 26 (92.9) 2 (7.1)	3.31 ± 1.6 44 (51.2) 42 (48.8)	3.05 ± 1.6 70 (61.4) 44 (38.6)	**0.000** **0.000**
Number of antenatal care visits 0 to 1 time 2 to 3 times ≥4 times	5 (18.5) 7 (25.9) 15 (55.6)	22 (26.2) 5 (6.0) 57 (67.9)	27 (24.3) 12 (10.8) 72 (64.9)	**0.014**
Time of breastfeeding initiation ≤1 h 1–23 h ≥24 h	8 (28.6) 14 (50.0) 6 (21.4)	27 (31.8) 20 (23.5) 38 (44.7)	35 (31.0) 34 (30.1) 44 (38.9)	**0.019**
Depression screening among mothers Minimal symptoms (PHQ-9 < 5) Mild depression (PHQ-9 5–9) Moderate to severe depression (PHQ-9 ≥ 10)	22 (88.0) 1 (4.0) 2 (8.0)	50 (60.2) 18 (21.7) 15 (18.1)	72 (66.7) 19 (17.6) 17 (15.7)	**0.032**
**Infant health characteristics**				
Suffered from symptoms in the past two weeks None Vomiting Irritability Diarrhea Fever Cough/Wheeze Runny nose/cold Insomnia Fatigue Ear infection	9 (32.1) 7 (25.0) 5 (17.9) 5 (17.9) 2 (7.1) 4 (14.3) 2 (7.1) 3 (10.7) 0 (0.0) 1 (3.6)	29 (34.5) 21 (25.0) 22 (26.2) 19 (22.6) 21 (25.0) 18 (21.4) 14 (16.7) 7 (8.3) 8 (9.5) 4 (4.8)	38 (33.9) 28 (25.0) 27 (24.1) 24 (21.4) 23 (20.5) 22 (19.6) 16 (14.3) 10 (8.9) 8 (7.1) 5 (4.5)	0.818 1.000 0.372 0.595 **0.043** 0.410 0.212 0.702 0.090 0.792
Received medicines in the past two weeks No Yes If yes, type of medicine Pain killers/Anti-inflammatory tablets Antibiotics	20 (76.9) 6 (23.1) 1 (16.7) 0 (0.0)	42 (53.2) 37 (46.8) 25 (67.6) 5 (13.5)	62 (59.0) 43 (41.0) 26 (60.5) 5 (11.6)	**0.033** **0.018** 0.338
Received any supplements in the past six months No Yes If yes, type of supplements received Vitamin D Multivitamins Iron	7 (25.9) 20 (74.1) 10 (55.6) 1 (5.6) 1 (5.6)	36 (42.4) 49 (57.6) 25 (52.1) 8 (16.7) 5 (10.4)	43 (38.4) 69 (61.6) 35 (53.0) 9 (13.6) 6 (9.1)	0.126 0.801 0.241 0.541
**Anemia and nutritional status of infants**				
Hemoglobin concentration (g/dL) Mean (±SD) 25th percentile 50th percentile (median)	11.70 ± 1.8 10.67 11.80	11.35 ± 1.1 10.50 11.40	11.43 ± 1.3 10.50 11.40	0.239
Anemia (Hb < 10.5 g/dL)	6 (23.1)	17 (19.8)	23 (20.5)	0.714
Stunting (L/HAZ < −2)	2 (7.1)	0 (0.0)	2 (1.8)	**0.012**
Underweight (WAZ < −2)	1 (3.6)	5 (5.8)	6 (5.3)	0.644
Wasting (WHZ < −2)	0 (0.0)	11 (12.8)	11 (9.6)	**0.046**
Overweight/obese (BAZ > +2)	1 (3.6)	5 (5.8)	6 (5.3)	0.539
Microcephaly (HCZ < −2)	2 (7.1)	3 (3.5)	5 (4.4)	0.412
Mid-upper arm circumference Z-score mean (±SD) Acute malnutrition (<115 mm) At-risk malnutrition (115–129 mm)	1.62 ± 1.0 2 (7.1) 6 (21.4)	0.23 ± 1.2 8 (9.3) 17 (19.8)	0.34 ± 1.3 10 (8.8) 23 (20.2)	**0.034** 0.931

PHQ-9: Patient Health Questionnaire-9; SD: Standard Deviations; Hb: Hemoglobin Concentrations; L/HAZ: Length/Height-for-Age Z-score; WAZ: Weight-for-Age Z-score; WHZ: Weight-for-Length/Height Z-scores; BAZ: Body Mass Index-for-Age Z-score; HCZ: Head Circumference Z-score; ^a^ Categorical variables are expressed as n (%) and continuous variables are expressed as mean ±SD. Lack of corresponding sum of frequencies with total sample size is due to missing data. ^b^ Significantly different at *p*-value < 0.05 (in bold).

**Table 4:** Key determinants of selected breastfeeding and child growth indicators among infants under six months.

Independent Variables	Early Initiation of Breastfeeding (WHO Indicator 2) ^a^ Reference Group: No (>1 h) ^b^	
	Unadjusted OR (95% CI)	Adjusted OR (95% CI)
Type of delivery Vaginal birth Caesarean section	1.0 (Reference) 0.39 (0.15; 0.99) *	1.0 (Reference) 0.85 (0.10; 1.43)
Self-reported gestational age (months) Full-term Preterm	1.0 (Reference) 0.27 (0.08; 0.98) *	1.0 (Reference) 0.18 (0.03; 0.98) *
Received pre-lacteal feeding No Yes	1.0 (Reference) 0.06 (0.02; 0.15) ^α^	1.0 (Reference) 0.06 (0.02; 0.20) ^α^
Weight-for-length/height Z-score	1.43 (1.04; 1.97) *	1.58 (1.03; 2.43) *
MDD-W of mothers Achieved (≥5) Not achieved (<5)	1.0 (Reference) 6.83 (2.20; 21.16) ^α^	1.0 (Reference) 5.52 (1.28; 20.75) *
Waist circumference of non-pregnant mothers ^c^ Normal At-risk	1.0 (Reference) 0.36 (0.15; 0.90) *	1.0 (Reference) 0.19 (0.05; 0.82) *
**Independent variables**	**Time of breastfeeding initiation ^a^** **Reference Group: No (<24 h) ^d^**	
	Unadjusted OR (95% CI)	Adjusted OR (95% CI)
WHO Indicator 3: Exclusive breastfeeding No Yes	1.0 (Reference) 0.34 (0.12; 0.92) *	1.0 (Reference) 0.60 (0.17; 2.05)
WHO Indicator 16: Bottle feeding No Yes	1.0 (Reference) 3.18 (1.45; 6.98) *	1.0 (Reference) 2.31 (0.92; 5.77)
Depression screening Minimal symptoms Mild depression Moderate to severe depression	1.0 (Reference) 2.65 (0.94; 7.45) 4.36 (1.43; 13.35) *	1.0 (Reference) 2.19 (0.74; 6.48) 3.46 (1.09; 10.97) *
**Independent variables**	**Exclusive breastfeeding (WHO indicator 3) ^a^** **Reference Group: No ^e^**	
	Unadjusted OR (95% CI)	Adjusted OR (95% CI)
Age of the infant (months)	0.62 (0.46; 0.85) *	0.56 (0.39; 0.81) *
WHO Indicator 16: Bottle feeding No Yes	1.0 (Reference) 0.03 (0.00; 0.20) **	1.0 (Reference) 0.04 (0.00; 0.38) *
Received breast milk substitutes instead or in addition to breast milk No Yes	1.0 (Reference) 0.12 (0.05; 0.32) ^α^	1.0 (Reference) 0.38 (0.11; 1.34)
**Independent variables**	**Weight−for−length/height Z−score ^f^**	
	Unadjusted β (95% CI)	Adjusted β (95% CI)
Age of the mother (years) <25 years ≥25 years	0.0 (Reference) 0.58 (0.07; 1.09) *	0.0 (Reference) 0.50 (−0.12; 1.12)
Received breastmilk substitutes: infant formula No Yes	0.0 (Reference) 1.32 (0.20; 2.44) *	0.0 (Reference) 1.41 (0.34; 2.48) *
WHO Indicator 3: Exclusive breastfeeding No Yes	0.0 (Reference) 0.63 (0.08; 1.18) *	0.0 (Reference) 0.64 (−0.08; 1.36)
WHO Indicator 16: Bottle feeding No Yes	0.0 (Reference) −0.73 (−1.23; −0.22) *	0.0 (Reference) −0.83 (−1.52; −0.14) *

WHO: World Health Organization; OR: Odds Ratio; MDD-W: Minimum Dietary Diversity for Women; * *p* < 0.05, ** *p* < 0.001, ^α^ *p* < 0.001; ^a^ All variables that have shown a *p*-value < 0.05 in the univariate logistic regression were included in the multiple logistic regression models. Adjusted Odds Ratio of the dependent variable are presented with 95% CI using multiple logistic regression. ^b^ The reference group for the dependent variable Early Initiation of Breastfeeding is No (>1 h) vs. Yes (≤1 h). ^c^ Non-pregnant mothers include lactating and non-pregnant non-lactating mothers. ^d^ The reference group for the dependent variable Time of Breastfeeding Initiation among infants is No (<24 h) vs. Yes (≥24 h). ^e^ The reference group for the dependent variable Exclusive Breastfeeding is No vs. Yes. ^f^ All variables that have shown a *p*-value < 0.05 in the univariate linear regression were included in the multiple linear regression models. Adjusted βs of the dependent variable for the Weight-for-length/height Z-score (as continuous dependent variable) are presented with 95% CI using multiple linear regression.

**Table 5:** Enablers and barriers for the initiation of breastfeeding and exclusive breastfeeding among Syrian refugee mothers of children under five years in vulnerable areas of Greater Beirut, Lebanon –qualitative analysis of focus group discussion (N = 11).

Themes	Codes	Representative Quotes
**A-** **Enablers for early initiation of breastfeeding and exclusive breastfeeding**		
Knowledge on infant feeding practices and maternal nutrition	Cultural beliefs	*“I wished I was able to breastfeed. They say that a child who has taken his mother’s milk develops a better immunity and becomes gentle and caring.” (Mother 1)* *“I gave him what he deserves.” (Mother 2)* *“I don’t want to stop breastfeeding, I want to give him his right to breastfeed, even if it’s from my heart, I want to give him his rights.” (Mother 3)*
	Maternal nutrition	*“Some people have certain convictions, that when the child breastfeeds from his mother, everything the mother eats comes with the milk, so him, he eats everything.” (Mother 4)* *“Now I eat everything, I’m breastfeeding the girl, I’m eating everything (…) I didn’t stop anything honestly.” (Mother 5)* *“We should not eat until we’re very full, so that the child doesn’t feel full. But we should eat and not hold back on anything.” (Mother 6)*
	Exclusive breastfeeding	*“[I gave my children] only breast milk, until six months (…), not even water.” (Mother 4)* *“[Just] my breast milk [since the beginning] (…), [with] nothing [else at all].” (Mother 7)* *“I started [feeding my child] at seven months (…), [in the beginning it was] only [breast] milk.” (Mother 8)* *“I breastfeed her only, I don’t give the bottle at all to my children.” (Mother 9)*
Support from healthcare providers and family	Healthcare professional support	*“My mother used to tell me to stop this and that, I didn’t listen to her, I know the doctor says we can eat everything, I listen to the doctor. (…) She would tell me, don’t eat hummus, don’t eat lentil, those bloats. But whatever the doctor tells me I listen to him. (…) and the doctor, when she gave me the C-section she told me to eat everything so that the child can get used to them [family food] later.” (Mother 5)*
	Family support	*“[When I am worried or tired], I can’t breastfeed (…), I wait till I calm down. (…) My husband’s sister [gave me this advice]. My mother isn’t here, but she has a telephone, so I also take information from her. (…) Yes true [that we ask relatives], if the condition progresses, we go to the doctor, but we ask relatives more.” (Mother 10)* *“[I take advice from] my mother-in-law, (…) [she doesn’t live with me], but she has experience, she’s tried it, if you look at the internet, everyone says something… and some of which are not true either, but the mother law has tried them before.” (Mother 11)* *“We all breastfeed.” (Mothers 12 and 13)*
**B-** **Barriers for early initiation breastfeeding of and exclusive breastfeeding**		
Pre-lacteal feeding	Oral rehydration solutions	*“When they are in the hospital, when they bring them to breastfeed, [they would have given him a] water that is called “mayit el ghalib” [referring to oral rehydration solutions].” (Mother 13)* *“I, it’s different from what the doctor gives… I personally didn’t give the child any cumin [or water and sugar] when he was small. After the first day, the doctor gave me a small box [referring to a type of feed] and said to give it to him the first few days, and after that the milk came in and I was breastfeeding.” (Mother 14)*
Infant feeding practices	Early introduction of solid, semi-solid, or soft foods (before the age of six months)	*“My children starting three to four months to taste whatever I cook so they won’t be disgusted by it when they grow up. Now, they eat anything I put in front of them. (…) Everything, whatever I cook. I put a small quantity on my finger and in his mouth.” (Mother 1)* *“Of course, I started; I fed him the first month. (…) [For example], Yogurt, fig jam, herbal tea.” (Mother 3)* *“Around five months, (…) [I started to introduce] fruits, like apples, I remove the skin, I squeeze it for juice, a quarter of a cup I mean.” (Mother 15)* *“At around four months, I mashed rice [with breast milk] and fed it to him. [I also gave him] banana for example… apple. (…) when he was four months old, I gave him those meals…” (Mother 16)* *“He’s eating since he was four months, I feed them yogurt, mashed rice…” (Mother 17)*
Infant feeding practices	Infant formula milk use	*“During the first period, I start using the baby bottle. As you know, milk is present abundantly in the village.” (Mother 1)* *“[I was breastfeeding him for six months] but with help, from the first month, I got help with the [infant formula] milk. (…) First three months, or four months, [it was] only breast milk… then I started helping them with the [infant formula] milk.” (Mother 4)* *“[I started giving her infant formula milk] around two months and a half, because she never got very full, so I got her formula milk.” (Mother 17)* *“In ten days, she will be four months old. (…) [She’s receiving] my milk and I got her [infant formula milk]. (…) I’ve been doing it for a month and ten days.” (Mother 18)*
	Water and/or herbal tea consumption	*“[He was only breastfed until four months but received] water, yes.” (Mother 17)* *“[He was only breastfeeding until five months, but] with water of course, I gave him [bottled] water.” (Mother 19)* *“I gave him just drops [of water] (…) from day 40 [after birth].” (Mother 18)* *“I gave him so much herbal tea, he suffered from dehydration, so I took him to the hospital. (…) [I gave him the chamomile tea] because I didn’t have any milk left to breastfeed.” (Mother 3)*
Maternal health reasons	Perceived lack of breast milk	*“I had five kids, and I didn’t breastfeed anyone of them. The reason is that I don’t have [any breast] milk.” (Mother 1)* *“He is the only one I had to buy milk for. And the rest I breastfed them, but him, he is the only one whom I couldn’t breastfeed because it was dry. (…) After I gave birth, I didn’t produce a lot of milk.” (Mother 3)* *“[I didn’t try to breastfeed after the delivery, because] I didn’t have any breast milk left.” (Mother 19)* *“The youngest girl I have, I didn’t get [breast] milk to give her… I didn’t get milk… (…) only 2–3 months… and the milk stopped.” (Mother 20)* *“There wasn’t any milk for him to breastfeed on… three days I would put him, I would breastfeed [him], he would start crying and crying.” (Mother 13)* *“[The oral rehydration solution is given to the infant at the hospital] when, for instance, the breast of the mother doesn’t produce milk, it remains for two days, and three days, until it starts producing milk.” (Mother 12)*
	Insufficient maternal dietary intake	*“I had no milk; it was dry from lack of nutrition.” (Mother 3)* *“I stopped because I lost a lot of weight, and I couldn’t breastfeed anymore. My nutrition… I didn’t have any vitamins to strengthen my milk… (…) it didn’t have vitamins, I wasn’t taking any vitamins to strengthen my milk, it was just regular food. And I lost a lot of weight…” (Mother 21)*
	Poor mental health	*“[When I am worried or tired], I can’t breastfeed (…).” (Mother 10)* *“My mother-in-law has diabetes and hypertension, and thyroid problems, and my husband’s sister is pregnant, and the owner kicked her out. Now one week she sleeps with us, one week at my husband’s brother’s house. And how am I supposed to handle all that, and I have five kids, and I’m ill, I’m take relaxants [anti-anxiety drugs].” (Mother 20)*
	Maternal illness	*“But if you have a problem, your milk won’t help him if you’re sick, the breast milk will be only like water.” (Mother 2)* *“Yes, the doctor told me it was from my milk. I got a fever, and I got worked up, then I got this bacterium. (…) Honestly, I always get bothered [and angry] and develop fever, but this is the first time my son gets this bacterium, he almost died.” (Mother 19)*
	Feeling tired	*“I’m getting tired when I’m breastfeeding, I’m very tired, my bones, they hurt. (…) I can’t stand up, when I finish breastfeeding, I feel beaten and dizzy…” (Mother 3)* *“When I was breastfeeding. I used to get very tired, so bit by bit I introduced them [my children] to food. It affected my body. (…) I stopped breastfeeding her [one of her children], she needs to suite her needs, so she can forget what she used to take. She’s giving her what she wants so she would forget the milk. Because the mother is getting tired, she is getting tired…” (Mother 20)*
Maternal health reasons	Complications during pregnancy and delivery	*“[I breastfed my son only] one month. (…) I gave birth by caesarian surgery, and the wound got infected. So, the doctor prescribed medication. So, due to the odor of the medication, he started vomiting.” (Mother 22)* *“In breastfeeding I’m not harsh on the child. As much as I eat and nurture myself, I tried with this child. (…) but this child is an exception, I gave birth to him through C-section (…) and I bled through the surgery…” (Mother 4)* *“My son, this one, didn’t breastfeed. (…) I didn’t have [any breast milk], I had during pregnancy… (…) complications, yes and bleeding, and I didn’t get any [breast] milk…” (Mother 23)*
	Premature delivery	*“I have never breastfed them, (…) they were born early, (…) they were born in the end of the 7th month.” (Mother 24)* *“[The child] was [receiving] both [breast milk and infant formula milk] … Ever since I got him, he was very small, he was tired and that was the situation.” (Mother 25)*
Social factors	Distribution of breastmilk substitutes	*“No, even in Syria I didn’t breastfeed. (…) Neither here [in Lebanon] nor there [in Syria]. On the contrary, here they gave me [samples of infant formula] milk. In Syria, we had cows nearby, so I used to feed her that.” (Mother 1)* *“They helped me a lot with the [infant formula] milk, I didn’t consider getting the milk but Dar-El-Fatwa [local non-governmental organization] got them for me. (…) I used to visit the doctor there she’s very good, and she would automatically take out the milk and hand it to me, every time I went to see her. (…) Yes, thank God, it was not bad, now when he grew up a little, the doctor told me, now sometimes… Every time they have some left, she tells me to take a pack [of infant formula milk]. If there aren’t any, I get Nido [brand of powdered cow’s milk] and they cause a lot of constipation… Well, the Nido, because it isn’t for his age, they told me that it’s not good… yes but I still give it to him (…)” (Mother 25)* *“[I started to give her a bottle of milk at four months] because in the hospital they taught her that. She stayed in the hospital for four days, they taught her on the milk bottle, so I started giving her…” (Mother 26)*
	Recommendations to use infant formula milk by healthcare professionals	*“I went to the pharmacist and explained the situation, that you can’t tell hand from foot, and she’s always lying down, the pharmacist said, “she died, does she still have her soul?” I said yes. So, she gave me infant formula milks (NAN and Nido) from the pharmacy, and the girl got better from the first week. Because of my situation, when I came to Lebanon, financially we weren’t good. Until he [my husband] worked, and thankfully things got better.” (Mother 20)* *“There wasn’t any milk for him to breastfeed on. For three days I would put him, I would breastfeed, he would start crying and crying. (…) They took him to the doctor; I told him this child hasn’t taken milk in three days. (…) He [the doctor] gave me the sugary water, they told me to give it to him, and then he gave me [infant formula] milk. [The child received] formula milk from the start.” (Mother 13)*
	Misinformation from mothers-in-law	*“My mother-in-law used to nag on me not to eat, I started eating in secret. Yes, it’s true. They say it affects my stomach and they tell me not to eat except potato and rice only. (…) Yes, my mother-in-law used to tell me. After that in 20 days, I didn’t have milk left. (…) I didn’t eat what I wanted to, so I lost my milk.” (Mother 20)* *“I also started eating potato and rice, potato, and rice, [it was my mother-in-law’s advice]. (…) It was wrong. I lost my milk, what can I do.” (Mother 6)* *“Her [the mother-in-law] advice is right, she’s an old woman and she’s given birth and she knows…but with my last child I didn’t get milk. I didn’t get milk but it’s not her fault, but she also told me to get her [the infant] starch, so I fed her starch until she [almost] died [and looked very sick], you wouldn’t know the girl’s hand from foot. I don’t know, she [my mother-in-law] told me [to get infant formula milk], so I got.” (Mother 20)*

## Data Availability

The dataset analyzed during this study is available from the corresponding author on reasonable request.
